# Accounting for quality: on the relationship between accounting and quality improvement in healthcare

**DOI:** 10.1186/s12913-015-0769-4

**Published:** 2015-04-23

**Authors:** Dane Pflueger

**Affiliations:** Department of Operations Management, Copenhagen Business School, Solbjerg Plads 3, 2000 Frederiksberg, Denmark

**Keywords:** Quality, Quality improvement, Accounting, Measurement, Patient survey

## Abstract

**Background:**

Accounting-that is, standardized measurement, public reporting, performance evaluation and managerial control-is commonly seen to provide the core infrastructure for quality improvement in healthcare. Yet, accounting successfully for quality has been a problematic endeavor, often producing dysfunctional effects. This has raised questions about the appropriate role for accounting in achieving quality improvement. This paper contributes to this debate by contrasting the specific way in which accounting is understood and operationalized for quality improvement in the UK National Health Service (NHS) with findings from the broadly defined ‘social studies of accounting’ literature and illustrative examples.

**Discussion:**

This paper highlights three significant differences between the way that accounting is understood to operate in the dominant health policy discourse and recent healthcare reforms, and in the social studies of accounting literature. It shows that accounting does not just find things out, but makes them up. It shows that accounting is not simply a matter of substance, but of style. And it shows that accounting does not just facilitate, but displaces, control.

**Summary:**

The illumination of these differences in the way that accounting is conceptualized helps to diagnose why accounting interventions often fail to produce the quality improvements that were envisioned. This paper concludes that accounting is not necessarily incompatible with the ambition of quality improvement, but that it would need to be understood and operationalized in new ways in order to contribute to this end. Proposals for this new way of advancing accounting are discussed. They include the cultivation of overlapping and even conflicting measures of quality, the evaluation of accounting regimes in terms of what they do to practice, and the development of distinctively skeptical calculative cultures.

## Background

Accounting—that is, standardized measurement, public reporting, performance evaluation and managerial control—has become increasingly central to efforts to improve the quality of healthcare. Seeking to know about, assure and improve the quality of care, governments and other authorities have been developing and extending accounting processes and devices into ever more aspects of medical organization [1-4]. In the UK National Health Service (NHS), as elsewhere, these efforts are now far reaching and include: the specification, standardization, and incentivization of quality metrics for general practitioners and other providers; the development and extension of the NHS Surveys program; the development of Patient Reported Outcome Measures and Quality-Adjusted Life Year metrics for effectiveness research; the requirement for all healthcare providers to produce annual Quality Accounts; and the development of quality improvement packages and interventions with accounting and measurement at the core such as the Productive Series and Continuous Quality Improvement^a^.

Such accounting infrastructure is consistent with international quality improvement and health policy literature, where, although it is acknowledged to be only the starting point for improvement [5], accounting infrastructure is argued to provide the essential preconditions for improvement possibilities. It is seen, for example, to be the “sine qua non of a high-performing healthcare system” ([6] p.23), “clearly the first step in improving the quality of care” ([7] p.613) and “a necessary condition if the health system is to be held properly to account by citizens and patients” ([8] p. 675).

Accounting successfully for quality, however, is increasingly shown to be more complex and problematic than typically assumed [6,9]. Reviews of quality measurement activities illuminate a variety of measurement challenges that limit the potential of quality improvement initiatives and even produce dysfunctional effects [10-13]. The challenges include difficulty specifying a workable definition of quality, challenges determining appropriate levels and locations of measurement, and problems interpreting the meaning and significance of change alongside the effects of case-mix and other factors [14-16]. They also include resistance to measurement from those being measured [17], the development in some jurisdictions of “target fatigue” [18], unwillingness of the public to use and trust in public measures [19], and gaming activities resulting in ‘target myopia’ [5], “hitting the target but missing the point” [20], or other even more insidious behaviors [21].

These important findings have been productively integrated into the current quality improvement literature. This literature now highlights, for example, the need to distinguish between measurement for improvement and assurance [6,22], the need for measures to be built upon robust clinical evidence [23], and the need for measures to be compelling and credible for their users [1,5,24].

These findings, however, also raise more significant questions about the dominant quality improvement paradigm and its reliance on accounting more generally. Reviewing the literature on measurement, Loeb [25] concludes that the costs of accounting might actually outweigh the benefits to improvement:

There is little agreement on the philosophy of measurement, on what to measure, on whether or how to adjust for what the patient brings to the clinical encounter and how data should be analyzed, how to report the data; and of course the ultimate decision relates to the value of measurement. Measurement adds new costs to the health care delivery system ([25], p.i5).

Authors also increasingly highlight that quality improvement interventions have seldom been subject to critical investigation and their effectiveness has not been rigorously demonstrated [26-29]. As such, authors such as Sheldon ([14], p.3) are challenging the mainstream measurement preoccupation itself, calling for the quality measurement “juggernaut” to be slowed and rethought altogether (see also [30,31]).

This paper aims to contribute to this debate about the role of accounting in quality improvement. It does this, firstly, by providing a synoptic overview of the specific and limited conception of accounting and measurement that is expressed in mainstream quality improvement debates and interventions, exemplified in the 2008 *Darzi Review* of the NHS. It then contrasts this understanding of accounting with the evidence about what accounting entails and how it is achieved, as advanced in the broadly characterized ‘social studies of accounting’ literature. Illustrative examples are used to document the existence of these different and conflicting understandings of accounting, and to highlight their potential significance in practice.

These examples are extracted from a study of quality measurement activities in the NHS undertaken by the author between January 2010 and July 2013. During this time, semi-structured interviews lasting between 30 and 90 minutes were undertaken with regulators (n = 3), policy analysts (n = 3), survey developers (n = 6), as well as quality improvement specialists (n = 6), senior administrators (n = 5), doctors (n = 3), and nurses (n = 13) in two large NHS Foundation trusts (located in London and the North of England), alongside historical archive investigations. In the two trusts, the author also undertook a total of approximately 50 hours of observations of trust-wide and localized quality and quality improvement activities relating to the production of quality accounts, quality improvement training, discussions of quality improvement intervention efforts, and similar activities identified during the interviews. The findings presented in this paper did not, methodologically speaking, emerge inductively from this field research. Rather, the data is simply employed to provide illustrations of some of the theories and themes advanced within the social studies of accounting literature^b^. These illustrations, as such, do not intend to prove or disprove these theories—there is plenty of existing debate related to these topics in the accounting literature (see e.g. [32])—but to illustrate their possible existence in healthcare and potential consequences and effects.

In the following section, the dominant quality improvement discourse in the UK and internationally is summarized and discussed in relation to findings from the social studies of accounting literature. Three points of difference between how accounting is understood in the quality improvement literature and what it has been shown to entail in the accounting literature and practice are highlighted in sections three, four, and five with accompanying illustrations drawn from the field research described above. These differences are that accounting does not just find things out, but makes them up; that accounting is not simply a matter of substance, but of style; and that accounting does not just facilitate, but displaces, control. Section six provides new ways to think about accounting, on the basis of the findings above, which might allow it to come closer to offering the quality improvements that are envisioned.

### Common conceptions of accounting in quality improvement

Although it is commonly believed that accounting and performance management are relatively straight-forward and unitary tasks, authors have highlighted that in fact there are many different logics and ways to understand, operationalize, and motivate them, resulting in very different varieties of accounting regimes and consequences [33,34]. In the mainstream quality improvement and health policy literature there are a variety of overlapping and often conflicting ambitions surrounding accounting, yet their underlying logic is increasingly articulated in a similar and distinctive way. Indeed, the assumptions, propositions, and aspirations of the “science of quality measurement” [17,35-37] and the “continuous quality improvement”^c^ movement [38-41] in healthcare have provided an extraordinarily stable conceptual underpinning for various ambitions since roughly 1985. Since that time, and spreading from the USA internationally, a very distinctive way of conceptualizing, articulating and advancing accounting for quality has emerged. Although this way of thinking about and using accounting is often only implicitly acknowledged, a synoptic overview of recent quality improvement literature, debates, and interventions shows it to have three distinctive characteristics.

First, this literature conceptualizes the problem of accounting as a matter primarily of uncovering or *capturing information,* as a camera might*,* through its ever more precise and accurate measurement. It suggests that measurement is a matter of simply better understanding the pre-existing and unvarnished reality of quality—of dividing it up into more precise domains (such as patient safety, patient experiences, and clinical effectiveness) or characteristics (such as structures, processes and outcomes) and then applying a variety of technical tools to isolate these things (see [15]). This suggests that, accurately defined, accounting for quality is a technical process related largely to the development of the camera itself. It projects accounting as a matter of developing sharper and better lenses: of adjusting for case-mix, establishing data quality, removing potential noise, establishing attribution, undertaking sophisticated factor analysis, and developing more refined and sophisticated data management systems [42-44].

Second, and relatedly, the quality improvement literature conceptualizes the process of capturing information through the refinement of measurement systems as a matter of applying a set of *timeless scientific principles*. These principles, borrowing from the natural sciences, equate accuracy with representational faithfulness. As such, this literature suggests that there is one right measure of every aspect of quality that can only be determined through the ever more rigorous application of scientific measurement principles, and it is for this reason that increasingly universal standards are developed and deployed ([45], c.f. [46]). These standards are seen to be the product of a unidirectional and unbroken path in pursuit of quality information, pursued from Nightingale’s efforts during the Crimean war until today (e.g. [47]).

**Figure 1:**
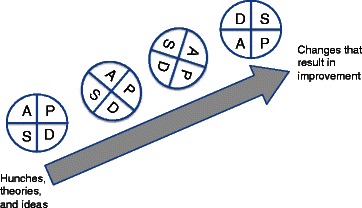
**The Plan, Do, Study, Act Cycle.**

This conception of accounting is characterized, thirdly, as inseparable from rationalized notions of performance management and managerial control [31]. Although Berwick and others have highlighted the extent to which information about quality for improvement and information for performance measurement and regulation are not the same ([38] p.634; [5]), the quality improvement literature suggests that one can only manage what one can measure. It implies that quality improvement can only be achieved through *finding and fixing*—that is, by accounting for quality and then making changes on the basis of these accounts alone [48]. This is highlighted in the now ubiquitous ‘Plan, Do, Study, Act’ (PDSA) cycle popularized by the Institute for Healthcare Improvement (Figure 1 below). It suggests that successful improvements are only achieved through measurements—through processes of measurement refinement that take you from “hunches, theories, and ideas” to “changes that result in improvement” [49]^d^.

The quality improvement literature, debates, and policies have, in summary, maintained a specific understanding of what accounting is, how it operates, and how it might be improved. This literature assumes that accounting is a technical and practical activity in which the ‘essence’ of quality is uncovered and revealed, and that these accounts comprise the only materials of management—the direct measures through which to find out what improvements are possible and how they can be done. Although this literature is critical of many ways in which measurements have been applied, it is largely non-reflective of its own assumptions about measurement—assumptions that have neither been explicitly debated nor shown to result in improvements [28,50].

Yet it is on the basis of these assumptions that seemingly ‘evidence-based’ quality improvement interventions and policies are consistently advanced and undertaken. Such assumptions, for example, underpin the UK’s recent and well-received quality improvement ambitions outlined in the 2008 *Darzi Review*. On the basis that “we can only be sure to improve what we can actually measure” ([51] p.49), the author (a high profile surgeon named Lord Darzi) outlined the steps of “bring[ing] clarity to quality”, “measur[ing] quality” and “publish[ing] quality” ([51] Ch.4) as central to achieving high quality care. These things were seen to be matters simply of standardizing, simplifying, and communicating the aspects of quality that already existed and that measurement science could illuminate. They were articulated as matters of “expanding the number and reach of national quality standards” ([51] p.49), providing a clearinghouse of robust and validated measures that clinicians and others could use to capture the reality of quality in any situation, and requiring providers to publish annual Quality Accounts “of the quality of their services, just as they produce financial accounts currently” ([51] p.25). These accounting activities were argued to lead naturally into the processes of “recognizing and rewarding quality improvement”, “raising standards”, “safeguarding quality”, and “staying ahead” ([51] Ch.4). With quality specified accurately, management was seen to be a matter of rewarding those measures, freeing professionals to interact with them, carefully monitoring core standards, and furthering education in quality improvement.

These accounting ambitions are built upon an evidence base that is more sophisticated than many of the target-based systems of improvement advanced in the UK in the past (see [20,52]). Yet, this research suggests, these accounting ambitions rest on a series of significant assumptions that run counter to a rich body of literature in an area broadly characterized as ‘social studies of accounting’^e^. This literature, most consistently developed in the journal *Accounting, Organizations, and Society*, attends to accounting not as a matter of more or less successful ‘implementation’, or of developing the ‘right’ measures, but as a social and institutional practice: a continually changing practice that is inseparable from both the aspirations toward which it is directed and the new realities that it creates [53-58]. Investigating the complex ways in which accounting interacts with its social and institutional environment, research in this field has shown the processes and activities of accounting to operate in ways quite different from those commonly understood and advanced.

Specifically, in contrast to the conceptions of accounting outlined in the quality improvement literature, the social studies of accounting literature shows accounting to be a fundamentally constitutive activity, creating as much as uncovering the phenomenon it seeks to reveal [59-61]. It shows the activities to produce seemingly ‘accurate’ accounts to be interconnected with particular political objectives and styles of knowing as much as representational faithfulness [62,63]. Finally, it shows strict reliance upon objective measures as a mechanism of management to produce undue comfort while displacing and creating risks of its own [64,65]. Each of these points are elaborated in the following sections, with accompanying illustrative examples drawn from the field research and related historical investigations described above.

### Accounting does not just find things out, but makes them up

Accounting, according to the improvement literature, is a secondary, derived, fact-finding activity—a matter of uncovering the essence of quality, which is seemingly timeless and universal. Those who study the historical and situated development of accounting systems, however, show the processes of accounting to be indistinguishable from the construction or ‘making up’ of the very things they seek to uncover or reveal [56,58,60,66-70]. These studies identify a variety of mechanisms of construction underlying the search for how things *really are*. They show processes of accounting, for example, to necessarily entail the imposition of a specific set of ambitions, preoccupations and objectives to make things know-able and account-able in a specific and often novel way [55,56,71,72]. They also show processes of accounting to take part in making these newly-configured people and things ever more ‘real’ social and organizational entities. By incorporating representations of people and things into systems of performance management and managerial control, they show, these representations can come to substitute for the things themselves [60,66,73]. This literature highlights, in other words, that the process of making things account-able makes up people and things through their incorporation and transposition into new networks and arrangements that establish “a particular way of understanding and acting upon” them ([59] p.1-2).

This constitutive aspect of accounting has been illustrated clearly in the case of cost. Of course, the notion of cost existed prior to the development of distinctive accounting technologies (such as standard, batch, and process costing systems) to find out what costs ‘really’ were. However, researchers show the way that the development of such systems took part in making up costs themselves. Miller and O’Leary [71], for example, showed the emergence of standard costing in the early years of the twentieth century to have involved the making up of a new notion of cost and new possibilities for the economic man. Standard costs, they explained, made costs into matters of efficiency and into something that could be attributed to every worker for the very first time. In this process, a new means of knowing about cost, and indeed a whole new economic reality was configured. Hopwood [66] illustrated a similarly constitutive process in the case of the early effects by Josiah Wedgewood to determine his costs of production in 1772:

Initiated to reveal what had been presumed to be there already, once established, it provided a basis for significantly changing, if not eventually transforming, the functioning of the enterprise. The newly established accounting system enabled a different set of dynamics to be set in motion […] The organization could be observed and managed in terms different from those in which it functioned. Attempts could be made to coordinate and plan different parts of the organization in the name of the economic. A quite specific organizational economy could start to be emergent ([66] p. 135).

Indeed, once accounted for, these authors show, costs and the people and things they are related to are never the same. They show, to paraphrase Ian Hacking, that representation and intervention move hand in hand [74].

One might question the proposition that quality too is made up in this way. Yet, activities to make quality calculable, like those to find out about costs, often bring quality into existence in healthcare in a particular and consequential way. This can be illustrated with reference to the emergence of “patient experiences” in the UK and elsewhere, as one essential measure of quality [44,75]. A short history of the emergence of a means to account for quality through patient experience surveys, such as the Picker survey in the UK and the Consumer Assessment of Health Providers and Services (CAHPS) survey in the USA, shows the way that accounting activities make up quality and even patients, and constitute them into healthcare reality in a novel way.

Indeed, experiences were crafted into one distinctive dimension of quality and one important aspect of healthcare practice though a specific urge to account that emerged in the late 1980s in the USA. At the time, a variety of changes in payment arrangements, healthcare costs, ideas about quality, expectations about care, and medical technologies were combining to make the calculation of quality an increasingly urgent preoccupation ([76]; c.f. [77]). Measures of quality were demanded, however, that extended beyond the traditional bio-medical outcomes of morbidity and mortality upon which discussions of quality were hitherto based, and included, at least in part, the patients’ view [78].

At the time, the measurement of “patient satisfaction” was advanced as a primary route through which to give expression to the patients’ view [79]. It was a measure that was precisely defined and measurable with a fair degree of accuracy [80,81]. Yet, it quickly became clear that this measure would not do. Although accounting was intuited to be a matter of uncovering the patients’ view, there were specific ideas about the ways in which this view needed to be made knowable. It was argued that this view had to be one that could differentiate between providers, specify what they did or did not do, and could provide actionable opportunities for improvement [39]. The satisfaction survey was inadequate for all these things because, among other things, it revealed *too much* about the patient, contaminating her view of what the providers did or did not do with her idiosyncrasies, moods, and whims [82].

To account for quality, therefore, a new perspective on the patient had to be forged. It was the technical work of the survey developer, John Ware, and his colleagues to ‘accurately’ account for the patients’ view that made up experiences as central to quality and in doing so transformed the dimensions of quality itself. Faced with these measurement challenges, he argued for the “rarely employed strategy” of measuring not satisfaction itself, but “patients’ reports of what does and does not occur” ([76] p.246). By breaking down satisfaction into a series of dimensions, and then, accessing these dimensions through carefully chosen questions about *patient experiences* with care, he argued, the patient’s view could finally be made account-able [83,84].

This type of accounting quickly became an accepted best practice for the measurement of the patient’s view and the new dimension of quality that it helped to create [85]. Hand in hand with the extension of patient surveys designed to “capture the specific experiences with care in terms of what did or did not happen from the consumer’s perspective” ([86] p.793), was the solidification of experiences as a core dimension of quality and means of expressing the patient’s view [87]. Accounting for quality thus became a matter of national survey programs asking not about general satisfaction, feelings, and perceptions, or about facts that the patients might observe, but about *experiences* such as how often “doctors *explained things* in a way that [the patient] could understand” [88].

Specific ideas about and technologies of accounting thus made up experiences as both a primary measure of quality and a means of patient expression in healthcare. In doing so, these accounting activities reconfigured, rather than merely revealed, the ‘reality’ of the patient and the boundaries of quality; both became matters of “experiences” as constituted within the questions of the patient experience survey. While experiences might have always had some relationship to quality and to medical care, efforts to more closely regulate, manage and reward quality, through mechanisms such as those outlined in the *Darzi Review*, are making experiences as constituted by the survey ever more central to the delivery of care. In the context of these efforts, providers are increasingly specifying “patient experiences” as a top organizational priority, they are installing systems to reproduce the survey throughout the organization at the ward level and in near-real time, they are allocating formal responsibility for experiences—sometimes to high profile Chief Experience Officers (CXOs)—and drawing from design and hospitality expertise in order to transform their organizations around the management of experiences (see [89-91]).

All of this activity, organized around experiences, is giving quality and indeed the patient a distinctive sort of life. Although the exact consequences of these changes is not yet clear, this illustration aims to show that accounting for quality is transforming both the reality of quality and the patient that it sought out to uncover. Whether these transformations are good or bad, the point of this illustration is that they are being brought about through the unacknowledged constitutive activities of accounting. As such, the assumption that measures, once deemed accurate, can be taken as undeniable reality is misleading. Rather, this section highlights that measurement accuracy represents the attainment of one of many possible realities, whose consequences and effects, rather than its assumed correspondence, should be the central focus of enquiry and debate.

### Accounting is not simply a matter of substance, but of style

The finding that measurement activities create as much as they discover has led a variety of researchers to enquire into the logics, rationales and “epistemic cultures” [92] that govern these constitutive measurement activities. What they have demonstrated, in contrast to the assumptions of the quality improvement literature, is that the terms of the seemingly scientific measurement enquiry are neither timeless nor immutable, but constituted by changing *styles* of enquiry [55,63,65,93,94]. These styles are the historically-specific ambitions, processes, and technologies through which accounting truth is sought. And these styles are shown not to correspond with a singular scientific method but merely one of many possible methods that is dominant or useful at the time [62]. These styles transform accounting and enquiry on the basis of changing ideas, ideals, and preoccupations operating in other diverse fields [55,70]. In transforming accounting, moreover, these styles shape the way in which the objects of accounting are made.

Michael Power illustrates the constitutive effect of style in the case of risk. He shows the way that, since the 1990s, risk has been transformed through accounting on the basis of the substitution of styles and infrastructure for making it up. He explains:

In a short period of time, the dominant discourse of risk management has shifted from the logic of calculation to that of organization and accountability. A statistical ‘empire of chance’ [...] which has developed over centuries […] has been rapidly subsumed within a new empire, namely that of the management control system ([65] p.4).

By replacing the terms through which risks are made account-able, Power shows, the reality of risks are transformed from calculations to systems, from possibilities to procedures, from threats to opportunities, and much else besides. Power and others thus highlight that the terms of good accounting change throughout time on the basis of changing preoccupations and concerns. It is therefore these styles of accounting that are central to making up the people and things that are accounted for in a particular way.

The centrality of style in accounting for quality can be seen in the example of the patient survey discussed above. Experiences did not emerge from the one and only form of enquiry available, for, at the time, authors advocated a number of avenues for separating the patient from the provider [95]. Rather, experiences were constituted through a unique arrangement of preoccupations and assumptions that converged at that particular point in time. These included the legitimization of cognitive psychological expertise for survey design [96], the emergence of the ambition not to know about patients but to know about what providers did for them [97], the propagation of a model of quality improvement which required that there were measurable things to improve [39], the redefinition of the patient as a consumer [98] and much else besides (see [99]). These movements and preoccupations, rather than measurement science as such, were the things that configured the terms through which the patients’ view was pursued and ultimately remade.

Style, however, is not simply a matter for measurement scientists. It also pervades accounting for quality at the more diffuse and localized levels of practice. Indeed, throughout healthcare practice and discourse, knowledge about quality is increasingly only produced in a specific way and on the basis of a specific style (c.f. [100,101]). This style is concerned with regulatory ambitions, political discourse, and ideals of control often at the expense of front-line and situated knowledge and practices. This is indicated in many of the practices to account for quality in NHS trusts observed in the years following the Darzi Review.

One of the major components of the review was the requirement for all NHS providers to produce annual Quality Accounts, documenting the quality of care they delivered in the areas of patient safety, clinical effectiveness, and patient experience. To do this, they were required to present a small number of indicators and a commentary about performance for each domain. Although it was stressed that the indicators should be selected locally on the basis of the aspects of quality that were relevant and meaningful [72,102], those tasked with compiling the reports quickly found that the terms of communicating knowledge about quality was constituted in a very specific way.

Although it is very likely that there is a wealth of information about quality that exists locally within each trust, the specified style for knowing about quality meant that ‘locally meaningful’ indicators of quality were simply those that aligned with external demands—that were specified in the operating framework, their commissioning contracts, and were used by regulators. In one trust studied by the researcher, the process of defining locally meaningful measures entailed simply the collation of those that were externally demanded. Evidence from the Kings Fund’s analysis of Quality Accounts suggests that others likely did the same. Instead of generating a diverse set of representation of quality at each trust, trusts produced Quality Accounts that were largely the same. Over 90 per cent of acute trusts, for example used the patient survey and hospital acquired infection rates as core measures ([102], p. 8).

The requirement for Quality Accounts to be externally audited further specified the style through which quality might be known. In the domain of patient experience, there are many ways to gain knowledge about quality such as through Executive ‘Walk Arounds’, patient diaries, Board Stories, exit interviews, monitoring of complaints and thank you cards, and surveys. However, the pursuit of a particular kind of objectivity through audit led trusts to systematically exclude all types of information about experiences other than that obtained through the survey. Auditors verified the information presented in Quality Accounts by comparing it to other data received by the Board [103]. In this context, it became important that the Board received fewer but more consistent forms of information about experiences. Board Stories and other sources of information became, according the Head of Quality Improvement and Safety at one of the trusts studied, “a bit of a liability to management” because the alternative realities of quality that they presented were a threat to the production of a seemingly robust account. Thus, the urge to account led to the enforcement of one style of knowing about quality at the expense of others, at least at the level of the Board.

These managerial styles of knowing, moreover, can filter down to the level of the ward and healthcare practice, thereby further displacing existing notions of quality. The demand for measures of quality for public reporting purposes almost always goes hand in hand with the development and extension of managerial ambitions to make these measures central to internal control systems (c.f. [64,72]). Measures of patient experience, clinical effectiveness, and patient safety used in Quality Accounts are incorporated into ward-level benchmarking activities, performance evaluations, reporting structures, and scorecards throughout the NHS.

The constitution, for example, of the national patient survey as synonymous with patient experiences has led some NHS trusts to install systems to reproduce the surveys throughout the hospital, and to measure the performance of individual wards in real time. As the Director of Organizational Development at one of the trusts studied explained of the organization’s response to the Darzi Review:

[…] Now we’ve got a quality report to the board, an executive lead for quality improvement, as well as governance and quality assurance. […] All these things are hugely positive. Now the organization can see the things that matter. The newsletter has the quality strategy in it each month. So I think the whole organizational outlook is changing.

Such managerial infrastructure allowed the Director there to proclaim, “we are measuring what matters now”. In doing so, however, these systems bring the external terms of knowing quality internally as well, overriding or displacing what might have existed before.

Front line staff that are subject to these managerial pressures, even if they maintain that quality is far more multidimensional than the accounts suggest, are constituted within a management accounting system that enforces one style of knowing about quality. They are made to understand their own performance through the measures and demand of their colleagues that they do the same. This is visible in many of the meetings and discussions that were observed in the aftermath of the Darzi reforms. During one meeting of nurses to discuss the new Intentional Rounding program that was being piloted as part of one trust’s effort to improve patient survey scores, for example, a Senior Nurse explained to her staff that although there seemed to be changes as a result of rounding, it was not measured, and therefore didn’t count. “There is no use just putting the [new processes] in without checking that its being used and that it is reliable and that the process is reliable” she explained. A Nurse Sister, nodding her head reiterated, “if it’s not documented it’s not done at all”.

Such stylistic measurement requirements transform the way in which quality might be understood and discussed in the sense that previous conceptions become idiosyncratic, unreliable, or just plain “hearsay”. As one senior physician explained to his colleagues at a training day for new consultants that the researcher observed, “this is why [the Quality Improvement facilitator] says measurement matters. Otherwise, he continued, you are relying on hearsay”. If not demonstrated reliably, he reminded his colleagues, you are “simply jumping to conclusions; something unfortunately that we in the NHS do too much”. Indeed, accounting for quality is a matter, as we have seen here, not of learning as much as possible about quality, but of standardizing and enforcing one style through which to know about particular aspects of quality, even if it might not yet be fully enforced.

In summary, although measurement and improvement activities often aspire to be locally and clinically led, they often specify distinctive terms through which this localized activity can be undertaken and communicated. Some managers, doctors, and nurses note this stylistic dominance. The directors with quality responsibilities at one trust studied, for example, explained much of their activities to account for quality as a matter of “feeding the beast, while still trying to do the right thing” and many nurses were quick to highlight that quality was something they understood by, for example, “getting a feel for the room”, or “putting themselves in their patients’ shoes”, rather than through the quality reports. However, this shift in the terms through which quality can be known, through its ever-growing managerial infrastructure, becomes increasingly difficult for practitioners to speak up against and show its limitations without being seen to be against quality.

### Accounting does not just facilitate, but displaces, control

The made up and therefore partial and selective nature of the things that are accounted for draws attention to the limitations of management by numbers and particular ‘find and fix’ ideas of control. Indeed, accounting scholars have highlighted the way in which accounting is increasingly coupled with rationalized forms of management—stressing control through the formalized specification of systems, procedures, and accountability mechanisms—to produce forms of intervention which are socially and politically comforting but ultimately displace and even exacerbate underlying problems and risks [72,104,105].

This sort of displacement of control through the expansion of mechanisms to manage through numbers has been illuminated starkly in the case of risk management in financial institutions. Authors highlight that the 2007 financial meltdown was predicated upon an unprecedented accumulation of risk made possible by the managerial and regulatory reliance upon institutionalized risk measurement and management models such as Value at Risk (VaR). These complex calculative tools specified one easily communicable and widely accepted but inherently limited risk measure. Ever greater attention to this measure and the management of risk strictly on the basis of the measure gave the impression that risks were contained while at the same time encouraging risks to be taken in the areas that VaR was unable to take into account [65,106-108]. Indeed, greater reliance on the measure led managers to “stuff risks into the tails”—they, in other words, built up risks in the areas of probability that VaR did not take into account ([106], np). These risks, and their consequences, however, ultimately became visible when those rare probabilities occurred. Rather than measuring and managing risks, institutional reliance on VaR created, we know with the benefit of hindsight, risks of its own.

Some of these same institutional conditions are visible in the management of quality and the pursuit of quality improvement in healthcare. By assuming that quality can be adequately and fully captured by numbers, and then managed through mechanisms of rationalized control, quality improvement efforts have the potential to displace quality. They, in other words, might control what is measured while encouraging the accumulation of poor quality in areas that the measures themselves hide. Indeed, such a possibility is visible in the management of patient experiences through the patient experience survey documented above. Although it is unclear the extent to which the reliance on this measure as a mechanism of managerial control creates greater risks of poor quality in areas not captured through the survey, it is certain that it creates a space of organizational unknowability where possibilities of risks and harms to patients may fester or grow.

Evidence of this displacement of control through management by numbers is accumulating through the emergence of almost continual quality failures and healthcare scandals right alongside the extension of managerial control infrastructure and ambitions. Indeed, this is one theme that has emerged from the 2013 *Francis Review* [109] of the quality failures at the Mid Staffordshire NHS Foundation Trust between 2005 and 2008. Francis characterizes the failures as in part due to the extension and elaboration of performance management and accounting regimes which failed to live up to the rationalized and cybernetic control principles that they were based upon. He explains that the Primary Care Trust (PCT) overseeing Mid-Staffordshire:

[was] under a duty to monitor and improve the quality of the services they commissioned. They were over time provided with tools which in theory would have enabled them to lay down safety and quality standards, monitor performance, and pursue remedies on behalf of patients, individually and collectively, where those standards had not been met. In general, however, the nationally available guidance did not lend itself to more than relatively crude measures in this regard, the focus remaining, as elsewhere in the NHS system, on financial control and a handful of access targets. Development of more sophisticated tools, both locally and nationally, was slow, with the result that it is not in the least surprising that, in spite of the rhetoric of quality, one of the worst examples of bad quality service delivery imaginable was not detected by this system. There was a significant gap between the theory of the PCTs role and their capacity to deliver ([109] p.48, 1.31).

Although Francis characterizes this failure of management as a matter of incomplete and imperfect development and implementation of management control, this can also be read as an outcome of the imperfect ideas and ideals of complete control through numbers. Indeed, the literature in the social studies of accounting suggests that the “gap between the theory of the PCTs role and their capacity to deliver” (ibid) might be due to the very limitation of accounting itself to accurately contain the elusive notion of quality in healthcare.

### Conclusions and recommendations

This paper has illuminated three distinctive ways in which ideas and ideals about how accounting operates and what accounting entails, as articulated in quality improvement literature and interventions, differ from the evidence provided in the social studies of accounting literature. This paper showed that the processes of accounting do not just find things out but also make them up; that accounting is not just about the underlying substance of its object, but the style through which that object is made known; and that management through accounting does not just control things, but displaces them to other locations where they might fester or grow.

These conceptual differences affect the way that care is delivered, and the way and extent to which quality improvement ambitions are achieved. This paper illustrated just some of the possible consequences that stem from the comforting but ultimately erroneous assumption that accounting for quality is a matter of capturing the preexisting and underlying essence of quality through the application of timeless and technical scientific principles. It showed that these assumptions, once carried over into practice, produce systems of measurement and management that generate less rather than more information about quality, that provide representations of quality which are oriented away from the reality of practice on the front line, and that create an illusion of control while producing areas of unknowability.

These findings problematize mainstream conceptions of accounting for quality improvement purposes. But they also offer insights into how accounting might be understood differently and indeed improved. This concluding section highlights the way that each of the three differences in the understanding of accounting outlined above offers alternative design principles for accounting systems which might more successfully achieve the ambitions of quality improvement.

Acknowledging that accounting makes things up as much as it finds things out provides a variety of new ideas for considering what an effective accounting system would entail. In particular, understanding accounting this way would caution strongly against the pursuit of the type of ever more centralized, standardized, and unified measures of quality that are common in policy discourse and interventions. Rather, a more ‘true’ or ‘precise’ accounting would be one that creates and contains many new, messy, overlapping, and always incomplete representations of quality.

Interestingly, the accumulation of legacy accounting systems in the NHS, as elsewhere, has provided some of the conditions for such messy, overlapping, and conflicting accounts to emerge, occasionally to productive effect [110]. In the mainstream quality improvement and public policy literature, the existence of such overlapping and contradictory systems, is seen to be a problem; it is seen in many situations to be an indictment of the seemingly disorganized state of quality measurement and improvement activities, and another reason for more streamlining, rationalization, and joined up approaches (see, e.g. [48,111]). The evidence presented in this paper, on the contrary, suggests that such overlapping systems offer an *opportunity* for quality improvement. It suggests that these systems, not despite but *because* they overlap and conflict, offers opportunities for learning more about what quality really is and how it really can be improved.

The realization of this opportunity, however, depends crucially on the ways in which these representations of quality are used and understood. This paper has shown that accounting is a matter of style as much as it is as matter of substance. This means that each representation of quality, and each legacy system of quality, performs and materializes a set of propositions about quality and its improvement. This highlights that each measure is not simply another, equally valid, perspective on quality, but a proposition about how a particular way of knowing quality might contribute to quality improvement. This also highlights that multiple and overlapping measures do not simply ‘add up’ to an ever more clear representation of quality. Indeed, to aggregate the measures by, for example, compiling them into some summary indicator as quality regulators often do (c.f. [112]), would be to ignore and overlook the propositions, theories, and politics that animate and are contained within the measures.

Rather, this paper suggests that different and overlapping measures can be best exploited through the advancement, evaluation, and critique of the *things that they do*. Indeed, rather than amalgamating ever more measures, this paper suggests the need to investigate, articulate, and evaluate the sorts of activities, actions, behaviors, and consequences that result from knowing about quality though a particular style or set of concerns. This would entail the development of an ever more fine-grained understanding of the complex ways in which different styles of accounting, manifest in different accounting systems or regimes, produce different effects in practice. In this way a more beneficial relationship between accounting and quality improvement could be learned, rather than assumed. This proposition is in contrast to the current debates about accounting, which suggest, tautologically, both that accounting is needed to improve quality, and that quality *is* what is accounted for. Such debates sidestep a central feature of accounting, which is that it performs a particular style for understanding of the world. Understanding this feature means that accounts should be discussed and evaluated not in terms of the accuracy they create, but in terms of what they achieve.

Finally, if we acknowledge that management by numbers displaces as much as it ensures control, then we might think of quality management not as a matter of operating on the basis of numbers, but of operating *around* numbers, and using them to show not what is known, but the boundaries of the unknown. Those studying risk management in banks offer useful lessons here. They showed that individual banks understood, interpreted, and responded to VaR calculations differently, leading to different outcomes. JP Morgan and other banks that were less damaged by the initial crisis, it has been shown, maintained a uniquely skeptical and responsive “calculative culture” [108] in which the VaR calculations were interpreted not as the underlying reality about risk, but as one of many possible indications of what risk might be [106]. These banks did not use the calculations as an “answering machine” [57], but instead used the changing numbers and trends to initiate discussions about what they might mean, what they might be missing, and what other calculations might be required.

The explicit development of these types of skeptical calculative cultures within the NHS, as elsewhere, might allow accounting to better serve the goals of quality improvement. Although the notion of culture is as elusive as quality, it is equally tangible in the sense of being constituted within specific regulatory systems or regimes. Indeed, calculative cultures of the wrong sort are already fostered and created through accounting regimes that urge and require certainty. These arguably could be turned around. Quality Accounts, for example, could be audited against the knowledge of nurses and doctors, rather than the measures themselves. Their narrative sections could summarize not just the quality of care that was delivered, but also what is unknown about the quality of this care. Further, the required improvement plans could relate to the capacity to know more about quality, rather than simply the capacity to address the aspects of it that are already known. Regimes of this sort encourage organizations to express, explain, and address the boundaries and possibilities of knowledge about and around quality, rather than focusing on the measures themselves.

This would arguably make the function of quality management a more difficult, uncertain, and complex task. Indeed, quality management would be a matter not of producing certainty, but uncertainty, ambiguity, and even organizational friction. It would also produce the risk that organized uncertainty, ambiguity, and friction might denigrate into mismanagement, irresponsibility, or even negligence. These tensions are neither irrelevant nor insurmountable (see [113]). However, it is the resolution of these tensions that this paper suggests might offer the greatest possibilities for accounting to contribute the goal of quality improvement that is so consistently shown to be required.

In summary, this paper adds to the accumulating evidence that existing practices of accounting for quality have a variety of dysfunctional and even counter-productive effects. However, it suggests that the call for accounting for the purposes of quality improvement to be abandoned or slowed is misplaced. It suggests that accounting is often ineffective not because it is inherently incomparable with quality and the complexities of healthcare, but because its underlying characteristics have not been fully acknowledged or understood.

This paper offered some ways in which the role of accounting for quality improvement might be reimagined on more theoretically and empirically sound terms. The new vision for accounting, while it is likely to provide the greatest potential for producing improvements, is a vision that, it must be acknowledged, does not provide the political benefits that existing accounting systems so perniciously do (c.f. [72]). Indeed, the new sorts of accounting systems that this paper envisions will not create illusions of certainty, accountability and control. Instead they will highlight the limitations of all these things: the boundaries of our knowledge, the complexities of accountability, and the impossibility of absolute control. As such, the strong case for politically unpalatable movements needs to be made—for real quality improvement might depend crucially upon these things.

## Endnotes

^a^In the USA, such initiatives include the National Commission for Quality Assurance’s (NCQA) HEDIS dataset, the Foundation for Accountability’s (FAACT) measurement instruments, and the Joint Commission’s ORXY database, among others.

^b^All identifiable information has been removed. Ethics committee approval was deemed unnecessary for this research.

^c^Blumenthal and Kilo (1998) explain: “CQI has its own distinctive characteristics. For one thing, CQI attempts to teach and promote the use of generic analytical methods that facilitate improvement in processes of all types, both clinical and nonclinical […] CQI is also distinguished by its promotion of managerial reforms that are designed to facilitate organizational change […] Westphal, Gulati, and Shortell (1997, 370) describe CQI as ‘an integrated management philosophy’. Central to this philosophy is a vision of leadership that encourages the creation of what Peter Senge has called ‘the learning organization’ […] Learning organizations promote the acquisition and use of new knowledge as central strategies for coping with the escalating complexity and continuous change in modern environments. Learning organizations also recognize the critical need to empower their workforces to learn and participate in continuous improvement.” (38. Blumenthal D, Kilo CM: **A report card on continuous quality improvemen**. *Milbank quarterly* 1998, **76**(4):625–648., p.627).

^d^This is also apparent in Berwick’s (1992) “eight principles of a system for improvement”. These include “1. Intention to improve, 2. Definition of quality, 3. Measurement of quality, 4. Understanding interdependence, 5. Understanding systems, 6. Investing in learning, 7. Reduction in costs, 7. Leadership commitment” (40. Berwick DM: **Continuous Quality Improvement in Medicine: From Theory to Practice: Heal thyself or heal thy system: can doctors help to improve medical care?***Quality in Health Care* 1992, **1** (Supplement):2.p.4).

^e^This field is closely connected with those of Science and Technology Studies (53. Sismondo S: **An introduction to science and technology studies**: John Wiley and Sons; 2011.), Social Studies of Finance (54. MacKenzie D: **Opening the black boxes of global finance**. *Review of International Political Economy* 2005, **12**(4):555–576.), and some strands of economic sociology (55. De Goede M: **Resocialising and repoliticising financial markets: contours of social studies of finance**. *Economic sociology: European electronic newsletter* 2005, **6**(3):19–28.; see; 56. Mennicken A, Vollmer H, A. P: **Tracking the Numbers: Across Accounting and Finance, Organizations and Markets**. *Accounting, Organizations and Society* 2009, **34**(5):619–637.; 57. Miller P, Power M: **Accounting, Organizing and Economizing: Connecting accounting research and organization theory**. *The Academy of Management Annals* 2013, **7**(1):555–603.).

## Abbreviations

NHS: National Health Service
PDSA cycle: Plan-Do-Study-Act cycle
CAHPS survey: Consumer Assessment of Health Providers and Services survey
CXO: Chief Experience Officer
VaR: Value at risk

## References

[1] Panzer R, Gitomer R, Greene W, Reagan Webster P, Landry K, Riccobano C (2013). Increasing Demands for Quality Measurement. J Am Med Assoc.

[2] Kaplan RS, Porter ME (2011). How to solve the cost crisis in health care. Harv Bus Rev.

[3] Shortell SM, Bennett CL, Byck GR (1998). Assessing the impact of continuous quality improvement on clinical practice: what it will take to accelerate progress. Milbank Q.

[4] Blomgren M, Sahlin K: Quest for Transparency Signs of a new institutional era in the health care field. In: *Transcending New Public Management.* edn. Edited by Christensen T, Lægrid P: Hampshire UK and Burlington VT: Ashgate; 2006.

[5] Berwick DM, James B, Coye MJ (2003). Connections between quality measurement and improvement. Med Care.

[6] Raleigh VS, Foot C: Getting the measure of quality. Opportunities and challenges. In*.* London: The King’s Fund; 2010.

[7] Epstein A, Smith P, Mossoalos E, Papanicolas I, Leatherman S (2009). Performance measurement and professional improvement. Performance Measurement for Health System Improvement: Experiences, Challenges and Prospects.

[8] Smith P, Mossoalos E, Papanicolas I, Leatherman S (2009). Performance Measurement for Health System Improvement: Experiences, Challenges and Prospects.

[9] Lohr KN (2004). Rating the strength of scientific evidence: relevance for quality improvement programs. Int J Qual Health Care.

[10] Lohr KM: Medicare: A Strategy for Quality Assurance, vol. 1. In*.* Washington, DC: National Academy Press; 1990.

[11] Shortell SM, Levin D, O’Brien J, Hughes E (1995). Assessing the evidence on Continuous Quality Improvement: Is the glass half empry or half full?. J Health Serv Admin.

[12] Blumenthal D, Epstein A (1996). Quality of Health Care. Part 6: The role of the physician in the future of quality management. N Engl J Med.

[13] Øvretveit J, Gustafson D (2002). Evaluation of quality improvement programmes. Quality Safety Health Care.

[14] Sheldon TA (2005). The healthcare quality measurement industry: time to slow the juggernaut?. Quality Safety Health Care.

[15] McGlynn EA (1997). Six challenges in measuring the quality of health care. Health Aff.

[16] Evans DB, Edejer T, Lauer J, Frenk J, Murray CJ (2001). Measuring quality: from the system to the provider. Int J Qual Health Care.

[17] Marshall MN, Shekelle PG, Leatherman S, Brook RH (2000). Public disclosure of performance data: learning from the US experience. Quality Health Care.

[18] Werner RM, Asch DA (2005). The unintended consequences of publicly reporting quality information. JAMA.

[19] Schneider E, Lieberman T (2001). Publicly disclosed information about the quality of health care: response of the US public. Quality Health Care.

[20] Bevan G, Hood C (2006). What’s measured is what matters: targets and gaming in the English public health care system. Public Adm.

[21] Llewellyn S, Northcott D (2005). The average hospital. Acc Organ Soc.

[22] Freeman T (2002). Using performance indicators to improve health care quality in the public sector: a review of the literature. Health Serv Manag Res.

[23] Chassin MR, Loeb JM, Schmaltz SP, Wachter RM (2010). Accountability measures—using measurement to promote quality improvement. New England J Med.

[24] Audet A, Doty M, Shamasdin J, Schoenbaum S (2005). Measure, learn, and improve: physicians’ involvement in quality improvement. Health Aff.

[25] Loeb JM (2004). The current state of performance measurement in health care. Int J Qual Health Care.

[26] Falconer JA, Roth EJ, Sutin JA, Strasser DC, Chang RW (1993). The critical path method in stroke rehabilitation: lessons from an experiment in cost containment and outcome improvement. Qual Rev Bull.

[27] Pellegrin KL, Carek D, Edwards J (1995). Use of experimental and quasi-experimental methods for data-based decisions in QI. Joint Comm J Quality Improve.

[28] Pronovost P, Lilford R (2011). A road map for improving the performance of performance measures. Health Aff.

[29] Kilpatrick KE, Lohr KN, Leatherman S, Pink G, Buckel JM, Legarde C (2005). The insufficiency of evidence to establish the business case for quality. Int J Qual Health Care.

[30] Braithwaite J, Wears RL: Resilient health care: Farnham UK: Ashgate; 2013

[31] Hollnagel E, Braithwaite J (2013). Wears R (eds.): Resilient health care.

[32] Boland RJ (1989). Beyond the objectivist and the subjectivist: learning to read accounting as text. Acc Organ Soc.

[33] Pollitt C (2013). The logics of performance management. Evaluation.

[34] Pollitt C, Harrison S, Dowswell G, Jerak-Zuiderent S, Bal R (2010). Performance regimes in health care: institutions, critical junctures and the logic of escalation in England and the Netherlands. Evaluation.

[35] Donabedian A (1988). The quality of care: How can it be assessed?. J Am Med Assoc.

[36] Donaldson MS (ed.): Measuring the quality of health care. Washington DC: National Academies Press; 1999. 25101409

[37] Chassin MR, Galvin RW (1998). The urgent need to improve health care quality: Institute of Medicine National Roundtable on Health Care Quality. J Am Med Assoc.

[38] Blumenthal D, Kilo CM (1998). A report card on continuous quality improvemen. Milbank Q.

[39] Berwick DM (1989). Continuous quality improvement as an ideal in health care. New England J Med.

[40] Berwick DM (1992). Continuous Quality Improvement in Medicine: From Theory to Practice: Heal thyself or heal thy system: can doctors help to improve medical care?. Quality Health Care.

[41] McLaughlin CP (2004). Continuous quality improvement in health care: theory, implementation, and applications.

[42] Consumer Assessment of Health Providers and Services, CMMS. Development of the Hospital Survey [https://cahps.ahrq.gov/surveys-guidance/hospital/about/Development-Hospital-Survey.html] Accessed June, 2014.

[43] Sequist T, Bates D, Smith P, Mossoalos E, Papanicolas I, Leatherman S (2009). Developing information technology capacity for performance measurement. Performance Measurement for Health System Improvement: Experiences, Challenges and Prospects.

[44] Edgman-Levitan S, Daley J, Delbanco TL (1993). Through the patient’s eyes: understanding and promoting patient-centered care.

[45] Veillard J, Garcia-Armesto S, Kadandele S, Lkazinga N, Smith P, Mossoalos E, Papanicolas I, Leatherman S (2009). nternational health system comparisons: from measurement challenge to management tool. Performance Measurement for Health System Improvement: Experiences, Challenges and Prospects.

[46] Timmermans S, Mauck A (2005). The promises and pitfalls of evidence-based medicine. Health Aff.

[47] Harrington L, Pigman H, Varkey P (2009). e. ie: Quality measurement. Medical quality management: theory and practice.

[48] World Health Organization: Quality of Care: A process for making strategic choices in health systems. In*.* Gneva: WHO Library; 2006.

[49] Institute for Healthcare Improvement, Plan-Do-Study-Act (PDSA) Worksheet [http://www.ihi.org/resources/Pages/Tools/PlanDoStudyActWorksheet.aspx] Accessed June 2014.

[50] Shojania KG, Grimshaw JM (2005). Evidence-based quality improvement: the state of the science. Health Aff.

[51] Darzi A: High Quality Care for All: Our Journey So Far. In*.* London: TSO; 2009.

[52] Mannion R, Davies H, Marshall M (2005). Impact of star performance ratings in English acute hospital trusts. J Health Serv Res Policy.

[53] Mennicken A, Vollmer H (2009). A. P: Tracking the Numbers: Across Accounting and Finance, Organizations and Markets. Acc Organ Soc.

[54] Miller P, Power M (2013). Accounting, Organizing and Economizing: Connecting accounting research and organization theory. Acad Manag Ann.

[55] Miller P, Hopwood A (1994). Accoutning as Social and Institutional Practice.

[56] Chapman C, Cooper D, Miller P (2009). Linking accounting, organizations, and institutions. Acc Organ Soc.

[57] Burchell S, Clubb C, Hopwood A, Hughes J, Nahapiet J (1980). The roles of accounting in organizations and society. Acc Organ Soc.

[58] Hopwood A (1978). Towards an organisational perspective for the study of accounting and information systems. Acc Organ Soc.

[59] Miller P, Miller P, Hopwood A (1994). Introduction. Accoutning as Social and Institutional Practice.

[60] Hines R (1988). Financial accounting: in communicating reality, we construct reality. Acc Organ Soc.

[61] Hopwood A (1987). The archeology of accounting systems. Acc Organ Soc.

[62] Porter T: Trust in numbers: The pursuit of objectivity in science and public life: Princeton MA: Princeton University Press; 1996.

[63] Samiolo R (2012). Commensuration and styles of reasoning: Venice, cost–benefit, and the defence of place. Acc Organ Soc.

[64] Power M (1999). The Audit Implosion: Managing Risk from the Inside. In.

[65] Power M (2008). Organized uncertainty: Designing a world of risk management.

[66] Hopwood A (1992). G: Accounting calculation and the shifting sphere of the economic. European Account Review.

[67] Morgan G (1988). Accounting as reality construction: towards a new epistemology for accounting practice. Acc Organ Soc.

[68] Chapman C, Cooper D (2012). Miller P (eds.): Accounting, Organizations and Institutions: Essays inHhonor of Anthony Hopwood.

[69] Miller P, O’Leary T (1993). Accounting expertise and the politics of the product: Economic citizenship and modes of corporate governance. Acc Organ Soc.

[70] Miller P, O’Leary T (1994). Accounting, ‘Economic Citizenship’, and the Spatial Reordering of Manufacture. Acc Organ Soc.

[71] Miller P, O’leary T (1987). Accounting and the construction of the governable person. Acc Organ Soc.

[72] Power M (1999). The audit society: Rituals of verification.

[73] Espeland WN, Sauder M (2007). Rankings and Reactivity: How Public Measures Recreate Social Worlds. Am J Sociol.

[74] Hacking I (1983). Representing and Intervening.

[75] Darzi A: Quality of Care for All. In*.* London: TSO; 2008.

[76] Office of Technology Assessment. The Quality of Medical Care: Information for consumers. In: Library of Congress (88–600537): US Congress. 1988.

[77] Scott R, Reuf M, Mendel P, Caronna C (2000). Institutional change and healthcare organizations: from professional dominance to managed care.

[78] Menninger WW (1975). Caring as Part of Health Care Quality. J Am Med Assoc.

[79] Cleary PD, McNeil BJ: Patient satisfaction as an indicator of quality care. *Inquiry* 1988;25(1):25–36. 2966123

[80] Leblow J (1974). Consumer Assessments of the Quality of Medical Care. Med Care.

[81] Locker D, Dunt D: Theoretical and methodological issues in sociological studies of consumer satisfaction with medical care. *Social Science and Medicine* 1978, Part A: Medical Psychology and Medical Sociology(12):283–292. 675282

[82] Pascoe GC (1983). Patient satisfaction in primary health care: a literature review and analysis. Evaluation Program plan.

[83] Ware JE, Snyder MK, Wright WR, Davies AR (1983). Defining and measuring patient satisfaction with medical care. Evaluation Program plan.

[84] Meng YY, Jatulis DE, McDonald JP, Legorreta AP (1997). Satisfaction with access to and quality of health care among Medicare enrollees in a health maintenance organization. West J Med.

[85] Gold M, Wooldridge J (1995). Surveying consumer satisfaction to assess managed-care quality: current practices. Health Care Financing Review.

[86] Weech-Maldonado R, Morales LS, Elliott M, Spritzer K, Marshall G, Hays RD (2003). Race/ethnicity, language, and patients’ assessments of care in Medicaid managed care. Health Serv Res.

[87] Delnoij DM (2009). Measuring patient experiences in Europe: what can we learn from the experiences in the USA and England?. European J Public Health.

[88] Consumer Assessment of Health Providers and Services Survey, CMMS. CAHPS Hospital Survey [http://www.hcahpsonline.org/home.aspx]

[89] Beryl Institute: Zeroing in on patient experience: Views and voices from the front line. In*.* Edited by Wolfe J: Bedford TX: Beryl Institute; 2010.

[90] Beryl Institute: A report on the Beryl Institute benchmarking study: The state of patient experience in American hospitals. In*.* Edited by Wolfe J: Bedford TX: Beryl Institute; 2011.

[91] Beryl Institute: The Chief Experience Officer: An emerging and critical role. In*.* Edited by Wolfe J. Bedford, TX: Beryl Institute; 2014.

[92] Knorr-Cetina K (1999). Epistemic Cultures: How the Sciences Make Knowledge.

[93] Desrosières A (2002). The politics of large numbers: A history of statistical reasoning.

[94] Ahrens T (1996). Styles of accountability. Acc Organ Soc.

[95] Linder-Pelz S (1982). Toward a theory of patient satisfaction. Soc Sci Med.

[96] Jobe J, Mingay D (1991). Cognition and survey measurement: History and overview. Appl Cogn Psychol.

[97] Ellwood P (1988). Outcomes management: A technology of patient experience. New England J Med.

[98] Mold A (2010). Patient Groups and the Construction of the Patient-Consumer in Britain: An Historical Overview. J Social Policy.

[99] Pflueger D (2013). Accounting for quality: the emergence and significance of managing for quality in healthcare. PhD Thesis Online.

[100] Zuiderent-Jerak T, Strating M, Nieboer A, Bal R (2009). Sociological refigurations of patient safety; ontologies of improvement and ‘acting with’quality collaboratives in healthcare. Soc Sci Med.

[101] Timmermans S, Berg M. The gold standard: The challenge of evidence-based medicine and standardization in health care: Philadelphia PA Temple University Press. 2003.

[102] Foot C, Raleigh V, Ross S, Tyscom T: How Do Quality Accounts Measure Up?: Findings from the First Year. In*.* London: The Kings Fund; 2011.

[103] Department of Health: Quality Accounts: 2011/12 audit guidance. DoH. London: TSO; 2012.

[104] Power M (2009). The risk management of nothing. Acc Organ Soc.

[105] Millo Y, MacKenzie D (2009). The usefulness of inaccurate models: Towards an understanding of the emergence of financial risk management. Acc Organ Soc.

[106] Nocera J (2005). The Quantitative, Data-Based, Risk-Massaging Road to Riches. In.

[107] Taleb NN: The Black Swan:The Impact of the Highly Improbable Fragility: New York NY: Random House LLC; 2010.

[108] Mikes A (2009). Risk management and calculative cultures. Manag Account Res.

[109] Francis R: Final Report Of The Independent Inquiry Into Care Provided By Mid Staffordshire NHS Foundation Trust. In: *The Mid Staffordshire NHS Foundation Trust Enquiry.* London: TSO; 2013.

[110] Van de Bovenkamp H, de Mul M, Quartz J, Weggellar-Jansen J, Marie A, Bal R (2014). Institutional layering in governing healthcare quality. Public Adm.

[111] Leatherman S, Sutherland K: The quest for quality: refining the NHS reforms: London: Nuffield Trust; 2008.

[112] Benzer M: Quality of life and risk conceptions in UK healthcare regulation: towards a critical analysis: Centre for Analysis of Risk and Regulation, London School of Economics and Political Science; 2011.

[113] Hood C, Jones DK: Accident And Design: contemporary debates on risk management: Abingdon UK: Routledge; 2003.

